# Narrative Review of High-Intensity Interval Training: Positive Impacts on Cardiovascular Health and Disease Prevention

**DOI:** 10.3390/jcdd12040158

**Published:** 2025-04-17

**Authors:** Jae-Myun Ko, Wi-Young So, Sung-Eun Park

**Affiliations:** 1 Department of Physical Education, Yonsei University, Seoul 03722, Republic of Korea; kjm1218@yonsei.ac.kr; 2 Department of Sports Medicine, College of Humanities, Korea National University of Transportation, Chungju-si 27469, Republic of Korea; 3 Department of Sport Science, University of Seoul, Seoul 02504, Republic of Korea

**Keywords:** high-intensity interval training, cardiovascular disease/prevention and control, exercise therapy, metabolic syndrome/rehabilitation, cardiorespiratory fitness

## Abstract

Background: High-intensity interval training (HIIT) has gained recognition for its positive impacts on cardiovascular (CV) health, metabolic outcomes, mental health, and quality of life (QoL). This narrative review aims to comprehensively evaluate the efficacy of HIIT in enhancing CV health and preventing CV disease (CVD). Methods: A comprehensive search of PubMed identified 257 articles, of which 39 studies met predefined inclusion and exclusion criteria for quality assessment. Key metrics evaluated included blood pressure, vascular function, lipid profiles, body composition, and CRF. Results: HIIT significantly improved vascular function, evidenced by reductions in systolic and diastolic blood pressure and enhanced flow-mediated dilation. Improvements in cardiac function were observed through increased cardiac output and heart rate variability. Additionally, HIIT positively influenced lipid profiles, decreasing low-density lipoprotein and triglycerides while increasing high-density lipoprotein. Significant reductions in body fat and improvements in VO_2_peak were noted, contributing to enhanced CRF. HIIT also positively impacted mental health and QoL, reducing anxiety and depressive symptoms. Importantly, HIIT was safely and effectively applied to high-risk populations—individuals with obesity, metabolic syndrome, CVD, and cancer survivors—with a low incidence of adverse effects. Conclusions: This review highlights HIIT as an effective and safe exercise modality for improving CV health, metabolic indicators, mental health, and QoL. Future research should focus on developing tailored HIIT protocols to optimize adherence and efficacy across diverse populations, considering variations in age, sex, health status, and underlying medical conditions.

## 1. Introduction

CVD encompasses a range of conditions, including heart disease, stroke, heart failure, and atrial fibrillation, and is the leading cause of morbidity and mortality globally [1,2]. In 2022, heart disease accounted for 702,880 deaths in the United States, representing one in every five deaths [2,3]. Given the rising global mortality from CVDs [4], CV research is critical. The American College of Sports Medicine (ACSM) advocates for increased physical activity as a cornerstone of health maintenance, highlighting regular exercise as vital for preventing weight gain and managing CVDs [5]. Specifically, exercise interventions aimed at reducing CVD risk promote fatty acid oxidation, improve insulin sensitivity, and induce beneficial vascular adaptations [4].

The intensity and frequency of exercise are key factors in CV protection [6]. Recently, high-intensity interval training (HIIT) has emerged as an effective exercise modality for promoting CV health and reducing CVD risk [7,8,9]. HIIT is defined as short bursts of intense physical activity performed at high exercise intensity loads—≥90% of running speed and >75% of maximal power output—aimed at ≥90% of an individual’s maximum oxygen uptake (VO_2_max) [10,11]. HIIT protocols vary and consist of different combinations of intensity and bout duration, such as long (2–4 min), short (5 s or less), sprint (20–30 s or more of maximal-intensity exercise), and repeated sprint intervals (10 s or less of maximal-intensity exercise), allowing for adjustments in the number of repetitions [12].

The literature predominantly highlights the positive effects of HIIT on cardiorespiratory fitness (CRF), vascular function, and metabolic adaptations [7,8,13,14,15]. Compared to moderate-intensity continuous training (MICT), it is more effective in reducing body fat mass and waist circumference, as well as improving CV function, despite shorter exercise durations [16,17]. Additionally, HIIT shows positive effects on the CV system, such as reducing blood pressure, improving vascular endothelial function, reducing arterial stiffness, and improving autonomic nervous system regulation [18,19,20]. These beneficial effects stem from distinct physiological mechanisms. For instance, HIIT enhances fat oxidation and lipolytic enzyme activity, contributing to reductions in body fat mass, particularly visceral adiposity [21]. It also improves vascular endothelial function and autonomic balance by increasing nitric oxide bioavailability and reducing arterial stiffness, thereby contributing to blood pressure regulation [22]. Furthermore, repeated high-intensity efforts stimulate mitochondrial biogenesis and increase stroke volume, improving VO_2_max and overall CRF [23]. Therefore, we propose that HIIT may induce physiological adaptations that efficiently regulate cardiac function, making it a promising strategy for preventing and managing CVDs.

HIIT has been widely applied across diverse populations and contexts. In healthy populations, including children and adolescents, HIIT has shown promising results in improving CV fitness, body composition, and metabolic health in controlled laboratory environments and practical settings such as school-based physical education programs [24]. In adults and older populations, HIIT has enhanced CV health, muscle strength, and functional fitness in recreational and clinical contexts [25]. Moreover, HIIT interventions have been extensively implemented among clinical populations, such as individuals with obesity, metabolic syndrome, and CVDs, as well as cancer survivors, demonstrating significant improvements in health-related outcomes [26,27,28,29,30]. Importantly, HIIT can be safely performed by high-risk populations, including individuals with cancer, metabolic diseases, hypertension, and coronary artery disease (CAD), improving CV health in the long term. Additionally, HIIT is a popular and effective training modality among competitive athletes, significantly enhancing performance parameters including aerobic capacity, anaerobic power, endurance, and recovery ability [31].

Although many studies have demonstrated favorable effects of HIIT on CV and metabolic outcomes, findings across the literature are not always consistent. Some trials have reported significant improvements in VO_2_max, blood pressure, and lipid profiles [26], whereas others have shown minimal or no effects [32]. These discrepancies may be attributed to variations in study design, sample size, population characteristics, and HIIT protocols, including differences in intensity, frequency, and duration. Given this heterogeneity, a comprehensive synthesis is essential to clarify the scope of HIIT’s effectiveness and its practical applications in CV health promotion and CVD prevention.

Despite the ongoing research into HIIT interventions across diverse populations, the previous literature reviews have often focused on specific groups or narrowly defined CVD prevention through HIIT. Although numerous clinical studies have documented HIIT’s efficacy in enhancing CRF, body composition, and biomarkers, there remains a scarcity of comprehensive reviews synthesizing its effects across various populations, including healthy older adults, individuals with obesity, metabolic syndrome, and high-risk patients. Given the increasing body of experimental research on HIIT, an integrative review of this nature is timely. Therefore, this study aims to comprehensively evaluate the effects of HIIT on CV health and CVD prevention. We systematically reviewed HIIT intervention studies to explore the physiological mechanisms underlying CV effects and their clinical applicability. The review underscores the necessity for personalized exercise prescriptions tailored to individual variations in exercise response.

## 2. Methods

### 2.1. Research Questions

This narrative review aims to address the following research questions:

(1) “What are the effects of high-intensity interval training (HIIT) on CV markers, including blood pressure, vascular function, lipid profiles, and metabolic outcomes?”
(2) “How does HIIT affect body composition and cardiorespiratory fitness (CRF) in diverse populations?”
(3) “Is HIIT a safe and effective intervention for improving CV and mental health, as well as quality of life across diverse health conditions, including high-risk groups?”

### 2.2. Search Strategy

This integrative review is based on the framework established by Whittemore and Knafl [33], comprehensively analyzing experimental studies that examine the effects of high-intensity exercise (including HIIT) on CV health. A literature search was conducted using PubMed, concluding on 10 February 2024. No restrictions were imposed on publication dates to ensure the inclusion of the widest possible range of studies. The search utilized Medical Subject Headings (MeSH) terms with the following strategy: (“High-Intensity Interval Training” [MeSH] OR “Exercise, High-Intensity Intermittent” [MeSH] OR “HIIT”) AND (“Cardiovascular Diseases” [MeSH] OR “Cardiovascular System” [MeSH] OR “Heart Disease Risk Factors” [MeSH]).

### 2.3. Eligibility Criteria

Inclusion and exclusion criteria were established to guide the literature screening. Eligible studies were limited to those with experimental designs investigating the impact of high-intensity exercise interventions on CV health. This included studies involving diverse populations such as healthy individuals, individuals with obesity, metabolic syndrome, CVD, and other chronic conditions, as well as cancer survivors, along with animal studies performing high-intensity exercise (including HIIT) over a specified period. Thus, participants varied broadly in age, sex, fitness level, health status, and underlying medical conditions. Moreover, studies reporting CV health-related outcomes were included. Priority was given to original research with experimental designs. We excluded studies focusing solely on single-bout exercise sessions, as well as review articles, systematic reviews, meta-analyses, conference abstracts, letters to the editor, protocols, and unpublished studies. Additionally, in vitro and ex vivo studies at the cellular level, as well as articles not published in English, were excluded. The selection process involved two independent researchers reviewing titles and abstracts to apply the eligibility criteria. Full texts were subsequently analyzed to finalize article inclusion. Disagreements were resolved through discussion.

### 2.4. Data Extraction and Study Reduction Strategy

The initial search yielded 257 articles, of which 138 met the preliminary screening criteria after applying inclusion and exclusion parameters. Given the large number of eligible studies, further screening was conducted using the methodology established by Whittemore and Knafl [33] to assess the quality of the studies. The quality assessment focused on study methodology reliability, design (randomized controlled trial [RCT], before-and-after comparative study, etc.), population characteristics (sample size, study setting, representativeness), the appropriateness of interventions, and the evaluation of CV health-related outcomes. Specifically, we excluded studies lacking clarity regarding the high-intensity exercise intervention or those with intervention protocols inconsistent with study objectives. Moreover, studies incorporating pharmacological or dietary interventions, case studies without control groups (follow-up and case studies), and those with small sample sizes were omitted based on quality assessment criteria. Each study was scored on a scale of 2 (excellent), 1 (fair), or 0 (excluded) for each criterion; only studies with a total score of 2 or higher were included. Two researchers independently assessed the studies, with disagreements resolved through discussion and consensus, yielding 39 articles for the analysis.

### 2.5. Data Analysis and Synthesis

Selected studies were systematically organized using Excel in Microsoft^®^ Office 2024 (Microsoft Corporation, Redmond, WA, USA) and analyzed for key characteristics and outcomes. Data were categorized by study design, HIIT intervention methods (intensity, frequency, and duration), participant characteristics (comorbidities, sample size, sex, and age), and CV health-related outcome variables (cardiac and vascular function, blood biomarkers, and body composition). The results are presented in the table to allow a comparison of study characteristics and key findings, providing a comprehensive overview of the effects of high-intensity exercise on CV health.

## 3. Results

### 3.1. Study Selection

**Figure 1:**
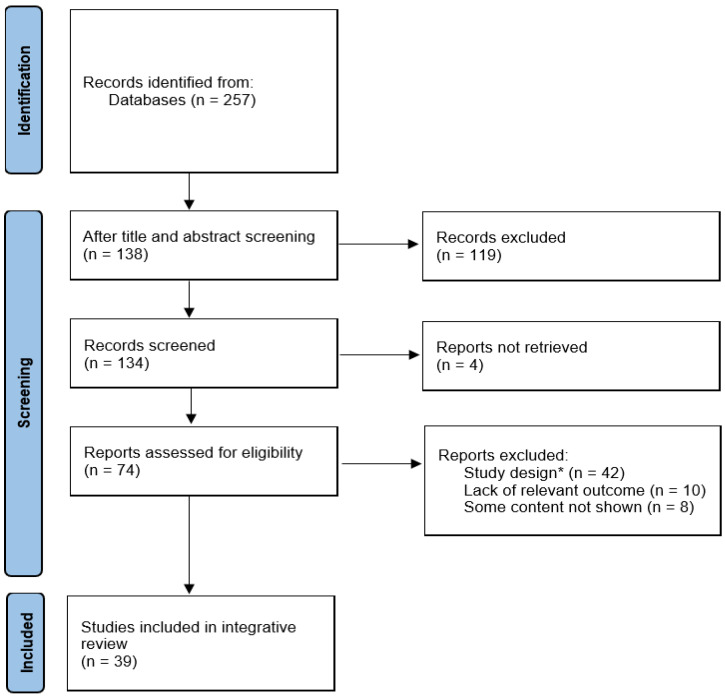
Flow diagram illustrating the study’s selection process. * study design criteria were employed to exclude studies with unclear high-intensity exercise protocols, single-session interventions, case and follow-up designs (without a control group), or extremely small sample sizes, as well as those incorporating drug interventions or dietary modifications. *n* = number.

A PubMed search identified 257 articles. After applying specific inclusion and exclusion criteria, 138 studies were selected for further evaluation. A quality assessment yielded 39 studies deemed suitable for inclusion in the analysis of the high-intensity exercise effects of high-intensity exercise on CV health. The study selection process is detailed in a flowchart (Figure 1).

### 3.2. Improvements in CV Markers

The included studies evaluated the effects of high-intensity exercise on various CV markers, including blood pressure, vascular function, heart rate control, blood lipid profiles, and metabolic health.

#### 3.2.1. Improving Vascular Function and Arterial Stiffness

HIIT reduced systolic (SBP) and diastolic blood pressure (DBP) [14,16,29,34]. HIIT improved vascular endothelial function by increasing FMD [8,17,20,35,36,37]. Decreased PWV and reduced peripheral vascular resistance have been observed, suggesting that HIIT lowers arterial stiffness and reduces CV burden [8,15,20,28,36].

#### 3.2.2. Improving Cardiac Function

HIIT positively influenced cardiac function and structure, resulting in increased left ventricular wall thickness and cardiac output, confirming its efficacy in improving cardiac function [9,25,35,38,39,40]. HIIT positively affected HRV, sympathetic nerve efficiency, and markers of cardiac injury [9,35,38,41].

#### 3.2.3. Improving Blood Lipid Profiles and Metabolic Health

HIIT decreased LDL, total cholesterol, and triglycerides (TGs), and increased HDL [7,8,13,28,32,41,42,43,44,45]. Additionally, it decreased hemoglobin A1c, increased insulin sensitivity, and improved fasting blood glucose levels, suggesting potential benefits for people with diabetes and metabolic syndrome [8,15,16,25,43,46,47,48]. Moreover, HIIT can improve intramuscular mitochondrial function, increase fat oxidation rates, and stimulate glucose metabolism [20,48].

### 3.3. Changes in Body Composition and Cardiorespiratory Function

The studies assessed the effects of HIIT on body fat loss, cardiorespiratory function, and muscle metabolism.

#### 3.3.1. Changes in Body Fat and Composition

HIIT has shown efficacy in reducing body fat mass and waist circumference [8,15,47,49].

Moreover, significant reductions in BMI and body fat percentage were reported [14,17,20,46].

#### 3.3.2. Improving CRF

Most studies have reported significant increases in VO_2_peak after HIIT interventions, with others also reporting improvements in CRF [8,9,13,14,17,20,25,27,28,30,37,39,40,42,44,45,46,47,49,50,51,52,53].

Increased CRF may play an important role in CVD prevention and health maintenance in various populations, including older adults, cancer survivors, individuals with obesity, and those with metabolic syndrome [25,27,32,44,49,52,54].

### 3.4. Other Factors

In addition to improving CV metrics and changing body composition, high-intensity exercise can affect exercise adherence, mental health, and autonomic nervous system regulation.

#### 3.4.1. Exercise Adherence and Safety

HIIT can be safely performed by high-risk populations, including individuals with CVD and metabolic syndrome, as well as cancer survivors, with most studies reporting minimal serious adverse effects [9,13,18,27,37,49,51,52,54]. However, an individualized approach is needed in high-risk patients, and exercise intensity and session frequency should be adjusted [53].

#### 3.4.2. Improving Mental Health and Quality of Life (QoL)

HIIT reduces stress, depression, and anxiety and improves QoL, particularly in those with CAD and cancer survivors [9,13,27,49,52].

#### 3.4.3. Individual Differences and Limited Effectiveness

HIIT did not significantly improve blood pressure or blood lipid profiles, with outcomes varying by age, sex, and health status [32,54,55]. Table 1 presents detailed information about the included studies.

## 4. Discussion

This narrative review comprehensively evaluated 39 studies analyzing the effects of HIIT on CV health and CVD prevention. Consistent with the previous literature, our study found that HIIT had positive effects across CV health indicators, including enhanced CRF (increased VO_2_max), vascular function (improved FMD), reduced blood pressure, and improved blood lipid profiles. The unique structure of HIIT—characterized by repeated high-intensity exercise followed by recovery—effectively induces cardiorespiratory adaptations and metabolic improvements, often outperforming MICT. These results suggest that HIIT can be utilized as a time-efficient strategy for promoting CV health. However, heterogeneity in study designs, participant characteristics, and exercise protocols needs to be considered when interpreting these findings. Overall, the studies exhibited similar trends, highlighting the positive effects of HIIT on CV health and functional indices. Jaureguizar et al. [27] reported that HIIT increased functional capacity (VO_2_peak and 6MWT) more than MICT and significantly improved QoL without increasing CV risk. This aligns with our findings, suggesting that HIIT is an effective strategy in cardiac rehabilitation and functional recovery.

HIIT involves repeated sessions of high-intensity exercise followed by low-intensity recovery and is effective in improving CRF in a short time [10,12]. A study by McGregor et al. [28] found that an 8-week HIIT program produced greater increases in VO_2_peak than MICT, indicating its role in improving CRF in the short term, aligning with our results. Collectively, our findings advocate for HIIT as an effective approach to improving CV function, particularly due to its time efficiency. MICT typically requires 30–60 min of sustained moderate-intensity exercise, whereas HIIT has shown equal or superior effects on CRF (VO_2_peak), vascular health (PWV and FMD), and blood pressure (SBP and DBP), despite shorter sessions [15,27,51]. This positions HIIT as a viable alternative for individuals with constrained schedules. However, the high-intensity nature of HIIT can be challenging for beginners and specific patient populations, necessitating the careful consideration of safety and sustainability [53,54]. In particular, high-intensity exercise may lead to rapid fatigue and increased musculoskeletal strain early in the exercise, potentially affecting exercise persistence and adherence [19]. In high-risk patients, the effects of HIIT may mirror those of MICT [53,54], suggesting the need to individualize exercise intensity. A gradual intensity increase following an adaptation phase, along with adequate recovery, may enhance safety and sustainability for these populations.

Long-term adherence strategies are also critical. McGregor et al. [51] found no significant differences between groups at a 12-month follow-up. A study by Berglund et al. [32] involved twice-weekly HIIT for 5 years, the longest intervention among the studies included in this review. They observed protective effects against HDL-C decline but no significant changes in other CV health markers. This suggests that long-term HIIT is limited in maintaining CV protection, highlighting the need for strategies to enhance exercise adherence and safety in the long term. Nevertheless, the results reviewed in this study suggest that HIIT is effective in high-risk populations, such as those with CVD and metabolic syndrome, and can be performed safely. HIIT improves cardiorespiratory function and vascular health in various high-risk populations, including those with CAD and cancer survivors, with a few serious adverse events [27,28,29,30]. Moreover, it enhances QoL and reduces stress, depression, and anxiety [18,42,52,53]. Therefore, HIIT may serve as a safe and useful exercise strategy for healthy adults and patients with CV and metabolic diseases. To optimize the long-term efficacy of HIIT, it is essential to minimize adverse effects and design tailored strategies that consider individual health conditions. Further research is needed to assess the long-term effects of modulating exercise intensity and frequency, adopting a stepwise approach to maintain adherence, and developing tailored HIIT protocols for high-risk populations.

The reviewed studies reported that HIIT is an effective exercise modality for improving CV health markers, though variations exist. VO_2_max, blood pressure, and blood lipid profiles were included as the primary indicators to assess CV health, whereas more nuanced measures of CV function—such as heart rate variability (HRV), flow-mediated dilation (FMD and endothelial function), and pulse wave velocity (PWV)—were considered in some studies. However, the limited or non-significant effects of HIIT have been found [29,55], potentially influenced by variables such as age, medical conditions, fitness levels, and the specifics of the HIIT protocols (intensity, frequency, and duration). For example, improvements in cardiorespiratory and vascular function were noted in healthy adults after just 6 weeks of HIIT [45], whereas individuals with conditions like diabetes or hypertension, as well as cancer survivors, required at least 8 weeks for significant changes [13,27]. Caution is warranted in interpreting results due to differences in study designs and methodologies. Variations in HIIT protocols (intensity, duration, and frequency) complicate direct comparisons between studies. Some studies used 4 × 4 min intervals [51], whereas others used 10 × 1 min intervals [16] or 30 s sprint repeats [8]. Moreover, the definition of “high-intensity” for HIIT varied across studies (e.g., VO_2_max 85–90%, HRmax, and RPE), making it difficult to compare results.

The diversity of the study populations, encompassing a range of demographics from healthy adults to those with CVDs and obesity, as well as older adults, poses challenges in assessing HIIT’s differential effects by sex, age, and fitness levels. For example, some studies included a male-dominated population, whereas others focused exclusively on postmenopausal women, limiting clear conclusions on sex and age differences. Moreover, most studies focused on short-term interventions, with a limited exploration of long-term CV health benefits. A study by Berglund et al. [32] involving HIIT performed twice weekly for 5 years did not show significant changes in CV health markers except for HDL-C increases. This suggests that HIIT is an effective way to improve cardiorespiratory and vascular function in the short term; however, moderating the frequency and intensity of exercise is needed to maintain long-term effects. However, long-term HIIT practice can cause muscle damage and fatigue accumulation, potentially reducing the longevity of CV health benefits [56,57,58]. Repetitive high-intensity training increases intramuscular calcium accumulation and triggers a chronic inflammatory response, leading to fatigue and muscle pathology [59]. Therefore, implementing adequate recovery periods and careful intensity management is crucial for the long-term viability of HIIT, necessitating further research in this area.

The substantial heterogeneity in study design and small population sizes across studies poses a challenge to drawing robust conclusions. These factors may have influenced the magnitude and consistency of the observed effects of HIIT. Therefore, caution is warranted in interpreting the findings. Several research challenges must be addressed to enhance the understanding of HIIT’s effectiveness and practicality. First, HIIT protocols need to be standardized. Variations in intensity, frequency, and duration, as well as differing definitions of “high intensity” across studies, including VO_2_max, HRmax, and RPE, complicate the comparability of study results and limit the ability to precisely test the effects of HIIT. Establishing a minimum set of common criteria will enable the consistent application of HIIT protocols in future research.

Second, studies should include more diverse populations. The predominance of small-sample (*n* < 50) studies and the restricted focus on specific populations (e.g., individuals with CVDs or women with obesity) limit the generalizability of findings to broader populations. To generalize the effects of HIIT and optimize its application, larger studies are needed that consider differences in age, sex, and health status.

Third, a significant gap exists in research on the long-term effects of HIIT. This limits our ability to determine whether the CV and metabolic benefits observed in the short-term studies are sustainable or clinically meaningful over time. Although short-term studies are useful to determine the immediate effects, long-term follow-up studies are essential to determine the sustained effects on CV health and disease prevention. For example, a study by Berglund et al. [32] showed no significant changes after 5 years of HIIT, except for an increase in HDL-C, indicating the need for strategies that enhance the long-term sustainability of HIIT. Factors such as exercise adherence, fatigue accumulation, and musculoskeletal strain are likely to play a role in the sustainability of HIIT effects and should be systematically studied.

Finally, a multidimensional evaluation approach is needed. Most studies have used key metrics such as VO_2_max, blood pressure, and blood lipids to assess the effects of HIIT; however, a precise assessment of CV function requires the inclusion of physiological markers such as HRV, FMD, and PWV. Furthermore, the effects of HIIT on metabolic health, muscle function, mental health, and QoL deserve further exploration.

In summary, despite existing methodological limitations, HIIT appears to be an effective and time-efficient strategy for improving CV health. However, individual variability in response to exercise and intervention duration underscores the necessity for ongoing research into the long-term applicability of HIIT and the design of tailored programs, which could increase exercise persistence and adherence and maximize its effectiveness. Establishing standardized protocols for HIIT and developing individualized exercise strategies—including periodic adjustments to frequency and intensity, as well as motivational approaches (e.g., group exercise or home-based programs)—for diverse populations will be important for future research and practical applications.

## 5. Conclusions

HIIT improves CV markers, body composition, and cardiorespiratory function and can be safely performed in high-risk populations (patients with cancer, metabolic syndrome, and CVDs). Furthermore, HIIT has been associated with improvements in mental health and QoL. However, an individualized approach is essential for high-risk groups, requiring adjustments to exercise intensity and session frequency. This narrative review provides the latest evidence supporting the effectiveness of HIIT across various populations and underscores its potential as a strategy for promoting CV health. Although all the studies included in our review had short durations, vigorous physical activity (exercise) interventions, including HIIT exercise conditions, the specific exercise protocols (e.g., intensity and session frequency) varied significantly. However, HIIT has demonstrated high effectiveness in enhancing CV health, with minimal reported adverse effects. Further research is warranted to optimize HIIT interventions tailored to individuals and diverse populations.

## Figures and Tables

**Table 1:** Studies on high-intensity interval training (HIIT).

Author, Year	Title	Study Type (Design)	(High-Intensity Interval Training) HIIT Protocol	Sample (Size, Sex, and Age)	Effect
Gonçalves et al., 2024 [16]	Effects of High-Intensity Interval Training vs. Moderate-Intensity Continuous Training on Body Composition and Blood Biomarkers in Coronary Artery Disease Patients: A Randomized Controlled Trial	RCT	6 wk, 4 × 4 min at 85–95% HRpeak, interspersed with 1 min recovery at 40% HRpeak, three sessions per week	Patients with coronary artery disease (CAD) (*n* = 69): HIIT (*n* = 23, 50 ± 9 y), moderate-intensity continuous training (MICT) (*n* = 23, 55 ± 10 y), and control groups (*n* = 23, 57 ± 11 y)	Vascular function (SBP and DBP), blood biomarkers (HbA1c, FBG, hs-CRP, TSH, T3, TC, TG, LDL-C, and HDL-C), and body composition (BFM and WC)
Nordén et al., 2024 [50]	Effect of high-intensity interval training in physiotherapy primary care for patients with inflammatory arthritis: the ExeHeart randomised controlled trial	RCT	12 wk of individualized HIIT at 90–95% HRpeak	Patients with inflammatory arthritis (*n* = 60, M/F)	Cardiac function (VO_2_peak)
Isanejad et al., 2023 [13]	Comparison of the effects of high-intensity interval and moderate-intensity continuous training on inflammatory markers, cardiorespiratory fitness (CRF), and quality of life (QoL) in breast cancer patients	RCT	12 wk, 4 × 4 min at 90% VO_2_peak, interspersed with 3 min active recovery at 50–60% VO_2_peak, three sessions per week	Patients with breast cancer (*n* = 30): HIIT (*n* = 10, 45.13 ± 6.86 y), MICT (*n* = 10, 45.29 ± 7.02 y), and control groups (*n* = 10, 44.97 ± 6.74 y)	Cardiac function (VO_2_peak), Blood biomarkers (HDL-C, SOCS3, and Estradiol), and others (QoL)
Rami et al., 2023 [38]	Highlighting the novel effects of high-intensity interval training on some histopathological and molecular indices in the heart of type 2 diabetic rats	Animal Study	8-week HIIT program, five sessions per week; started at 80% of peak speed in the first week, with a 10% increase in speed each week	32 male rats: healthy control (*n* = 8), diabetes control (*n* = 8), diabetes training (*n* = 8), and healthy training groups (*n* = 8)	Cardiac function (↓pathological hypertrophy, ↓fibrosis, ↓apoptosis, ↓β-catenin, ↓c-Myc, ↑GSK3B, and ↑Bcl-2)
Taha et al., 2023 [7]	Effect of high intensity interval training on arterial stiffness in obese hypertensive women: a randomized controlled trial	RCT	12 wk, 4 × 4 min cycling at 85–90% peak HR, interspersed with 3 min active recovery at 60–70% peak HR, three sessions per week	Women with obesity and hypertension (*n* = 60): HIIT (*n* = 25, 48.32 ± 4.48 y) and control groups (*n* = 28, 48.92 ± 3.60 y)	Vascular function (arterial stiffness, SBP, and DBP) and blood biomarkers (TC, TG, LDL-C, and HDL-C)
McGregor et al., 2023 [51]	High-intensity interval training in cardiac rehabilitation: a multi-centre randomized controlled trial	RCT	8 wk, two sessions/week; 10 × 1 min intervals at >85% peak power output (PPO), interspersed with 1 min recovery at 20–25% PPO (cycle ergometer); intensity increased biweekly if rating of perceived exertion (RPE) <17	Patients with stable CAD (*n* = 382, M/F, 18–80 y): HIIT (*n* = 187) and moderate- intensity steady state groups (*n* = 195)	Cardiac function (VO_2_peak)
Tang et al., 2022 [8]	Effects of aquatic high-intensity interval training and moderate-intensity continuous training on central hemodynamic parameters, endothelial function and aerobic fitness in inactive adults	Pre–post training intervention	6 wk, three sessions/week; 15 min land warm-up, 10 min aquatic warm-up, 12 × 30 s breaststroke bouts at 95% HRmax (RPE 15–18/20) with 60 s rest between bouts; 10 min cool-down	Inactive adults (*n* = 26, M = 7, F = 19): HIIT (*n* = 13, 42.2 ± 5.7 y) and MICT groups (*n* = 13, 39.2 ± 6.0 y)	Cardiac function (VO_2_peak and RHR), vascular function (endothelial function, SBP, and DBP)
Domaradzki et al., 2022 [14]	Prevalence of Positive Effects on Body Fat Percentage, CV Parameters, and CRF after 10-Week High-Intensity Interval Training in Adolescents	Pre–post training intervention	10 wk, one session/week; 10 min warm-up (jogging and stretching), three cycles of 8 × (20 s work/10 s rest) Tabata protocol, 1 min rest between cycles; various bodyweight exercises (push-ups, lunges, etc.)	Adolescents (*n* = 141, M = 52, F = 89): experimental (*n* = 73, M = 31, F = 42) and control groups (*n* = 68, M = 21, F = 47); mean age 16 y (M: 16.24 ± 0.34, F: 16.12 ± 0.42 y)	Vascular function (SBP and DBP) and body composition (BMI)
Domaradzki et al., 2022 [14]	The Mediation Role of Fatness in Associations between CRF and Blood Pressure after High-Intensity Interval Training in Adolescents	Pre–post training intervention	10 wk, one session/week; 10 min warm-up (jogging and stretching), three cycles of 8 × (20 s work/10 s rest) Tabata protocol, 1 min rest between cycles	Adolescents (*n* = 64, M = 28, F = 36, 16 y)	Vascular function (SBP)
Hovsepian et al., 2021 [17]	The Effect of All Extremity High Intensity Interval Training on Athero-Protective Factors and Endothelial Function in Overweight and Obese Women	Pre–post training intervention	10 wk, four sessions/week; 4 × 4 min at 85–90% HRmax interspersed with 3 × 3 min recovery at 70% HRmax; 40 min/session	Women with overweight status or obesity (*n* = 30, F): HIIT (*n* = 15) and control groups (*n* = 15); 20.53 ± 1.50 y	Cardiac function (VO_2_max), vascular function (FMD), blood biomarkers (adiponectin), and body composition (weight, WC)
Haglo et al., 2021 [52]	Smartphone-Assisted High-Intensity Interval Training in Inflammatory Rheumatic Disease Patients: Randomized Controlled Trial	RCT	10 wk, two sessions/week; 6 min warm-up at ~70% HRmax; 4 × 4 min intervals at 85–95% HRmax with 3 min active recovery at ~70% HRmax; Total session ~34 min	Patients with inflammatory rheumatic diseases (*n* = 40, F = 33, 48 ± 12 y, M = 7, 52 ± 11 y); supervised (*n* = 20) and app-guided groups (*n* = 20)	Cardiac function (VO_2_max) and others (QoL)
Berglund et al., 2021 [32]	The Long-term Effect of Different Exercise Intensities on High-Density Lipoprotein Cholesterol in Older Men and Women Using the Per Protocol Approach: The Generation 100 Study	RCT	Two sessions/week for 5 y; 10 min warm-up at ~70% HRpeak; 4 × 4 min intervals at ~90% HRpeak with 3 min active recovery at ~70% HRpeak; 5 min cool-down at ~70% HRpeak	Older adults (70–77 y, *n* = 673, F = 350, M = 323): HIIT (*n* = 119); MICT (*n* = 142) and control groups (*n* = 412)	Blood biomarkers (HDL-C)
Vidal-Almela et al., 2021 [42]	Sex differences in physical and mental health following high-intensity interval training in adults with CVD who completed cardiac rehabilitation	Pre–post training intervention	10 wk, two sessions/week; 4 × 4 min at 85–95% HRpeak, 3 × 3 min at 60–70% HRpeak; total session: 25 min	Adults with CVD post-rehab (*n* = 140, F = 40, M = 100, 58 ± 9 y)	Cardiac function (VO_2_peak), blood biomarkers (TC, LDL-C, and HDL-C), and body composition (WC)
Bell et al., 2021 [53]	Additional CV fitness when progressing from moderate- to high-intensity exercise training in previously trained breast cancer survivors	RCT	12 wk, two sessions/week; cycle ergometer: first 2 wk: 4 × 2 min at 70–75% HRR, 2 min active recovery(60% HRR); next 10 wk: 4 × 5 min at 70–75% HRR, active recovery at 60% HRR	Survivors with breast cancer (*n* = 20, F): HIIT (*n* = 10) and MICT groups (*n* = 10); 35–60 y	Cardiac function (↑VO_2_peak and ↑MV) and body composition (↓WC)
Ghram et al., 2021 [9]	High-Intensity Interval Training in Patients with Pulmonary Embolism: A Randomized Controlled Trial	RCT	8 wk, three sessions/week; treadmill and cycle ergometer: 4 × 2 min at 80–90% HRpeak, 2 min active recovery (50–70% HRpeak)	Patients with intermediate-high risk PE (*n* = 24, M/F): HIIT (*n* = 12) and control groups (*n* = 12); 49.6 ± 13.6 y	Cardiac function (↑VO_2_max, ↑FEV1, and ↓RV/LV ratio) and others (↑QoL)
Edwards et al., 2021 [34]	Ambulatory blood pressure adaptations to high-intensity interval training: a randomized controlled study	RCT	4 wk, three sessions/week, cycle ergometer: 3 × 30 s maximal sprints (7.5% bodyweight resistance) with 2 min active recovery	Physically inactive adults (*n* = 41, M/F): HIIT (*n* = 21) and control groups (*n* = 20); 22.8 ± 2.7 y	Vascular function (↓SBP and ↓DBP)
Toohey et al., 2020 [18]	The impact of high-intensity interval training exercise on breast cancer survivors: a pilot study to explore fitness, cardiac regulation and biomarkers of the stress systems	Pilot RCT	12 wk, three sessions/week, stationary cycling: 7 × 30 s intervals (all-out effort) at 95–115 RPM, with 2 min active recovery; gradually increased from 4 to 7 intervals by week 4	Survivors of breast cancer with a sedentary lifestyle (*n* = 17, F): HIIT (*n* = 6), continuous moderate-intensity training (*n* = 5), and control groups (*n* = 6); 50–75 y	Cardiac function (↑VO_2_peak)
Way et al., 2020 [15]	The effect of low-volume high-intensity interval training on CV health outcomes in type 2 diabetes: A randomised controlled trial	RCT	12 wk, three sessions/week, 1 × 4 min cycling at 90% VO_2_peak, 10 min warm-up, and 5 min cool-down (total: 19 min/session)	Inactive adults with type 2 diabetes and obesity (*n* = 35, M/F): HIIT (*n* = 12), MICT (*n* = 12), and control groups (*n* = 11); 18–65 y	Cardiac function (↑VO_2_peak), vascular function (↓SBP and ↓PWV), blood biomarkers (↓Hb1Ac), and body composition (↓WC)
Farahati et al., 2020 [43]	The Impact of High-Intensity Interval Training Versus Moderate-Intensity Continuous Training on Carotid Intima-Media Thickness and Ankle-Brachial Index in Middle-Aged Women	Quasi-experimental (Pre–post intervention)	12 wk, three sessions/week, 4 × 4 min at 85–95% HRmax, and 3 min recovery at 50–60% Hrmax	Women with inactivity and overweight status (*n* = 30, F): HIIT (*n* = 10), MICT (*n* = 11), and control groups (*n* = 9); 40–50 y	Vascular function (↓CIMT and ↓right ABI) and blood biomarkers (↓TC and ↓TG)
Ramez et al., 2020 [41]	High-intensity interval training increases myocardial levels of Klotho and protects the heart against ischaemia-reperfusion injury	Animal Study	5 days of treadmill running: 6 × 2 min at 85–90% max capacity, 2 min recovery at 50–60%, with warm-up and cool-down at 40–50%	Male Wistar Rats (*n* = 70, M)	Cardiac function (↓infarct size, ↓CK-MB, ↓LDH, and ↓cTnI)
Arboleda-Serna et al., 2019 [55]	Effects of high-intensity interval training compared to moderate-intensity continuous training on maximal oxygen consumption and blood pressure in healthy men: A randomized controlled trial	RCT	8 wk, three sessions/week; 15 × 30 s sprints at 90–95% HRmax, 60 s recovery at 50–55% VO_2_max(treadmill)	Healthy men with inactivity (*n* = 44, 18–44 y)	No significant change
Scott et al., 2019 [20]	Home-hit improves muscle capillarisation and eNOS/NAD (P) Hoxidase protein ratio in obese individuals with elevated CVD risk	RCT	12 wk, three sessions/week; repeated 1 min bouts of bodyweight exercises with no rest between two 30 s exercises, followed by 1 min rest; target ≥80% HRmax	Individuals with obesity and ≥2 CVD risk factors (*n* = 32, M/F); home-HIT (*n* = 9), home-MICT (*n* = 13), and Lab-HIT groups (*n* = 10)	Cardiac function (↑VO_2_peak), vascular function (↑capillary density, ↑FMD, and ↓aortic PWV), body composition (↓BMI), and muscle function (↑mitochondrial density, ↑GLUT-4, and ↑IMTG)
Verboven et al., 2019 [35]	High intensity training improves cardiac function in healthy rats	Animal Study	13 wk, five sessions/week; 10 bouts of treadmill running (18 m/min, 30° incline) separated by 1 min of active rest	Healthy male Sprague Dawley rats (*n* = 26, M); HIIT (*n* = 8), MIT (*n* = 8), and control groups (*n* = 10)	Cardiac function (↑EF, ↓ESV, ↑SV, ↑AWT, and ↑PWT) and vascular function (↑capillary density)
Zhang et al., 2019 [39]	CV response of postmenopausal women to 8 wk of sprint interval training	RCT	8 wk, three sessions/week; 20 min/session (8 s sprints at near-maximal exertion (100–120 RPM) + 12 s recovery); performed on a cycle ergometer	Postmenopausal women with overweight status (*n* = 30, F): SIT (*n* = 15) and control groups (*n* = 15); 47–59 y	Cardiac function (↑VO_2_max, ↑SV, ↑DFT, and ↓RHR)
Ramírez-Vélez et al., 2019 [36]	Effectiveness of HIIT compared to moderate continuous training in improving vascular parameters in inactive adults	RCT	12 wk, three sessions/week; 4 × 4 min intervals at 85–95% HRR, 4 min recovery at 75–85% HRR; treadmill-based walking/running; total session time: 38–42 min	Adults with inactivity (*n* = 21): HIIT (*n* = 11) and MCT groups (*n* = 10); 18–45 y	Vascular function (↑brachial artery diameter and ↓PWV)
Hwang et al., 2019 [54]	Effect of all-extremity high-intensity interval training vs. moderate-intensity continuous training on aerobic fitness in middle-aged and older adults with type 2 diabetes: A randomized controlled trial	RCT	8 wk, four sessions/week; 4 × 4 min at 90% HRpeak, 3 × 3 min active recovery at 70% HRpeak; total session time: 40 min	Individuals with type 2 diabetes mellitus (T2DM) (*n* = 58, M/F): HIIT (*n* = 23, 65 ± 2 y), MICT (*n* = 19, 62 ± 2 y), and control groups (*n* = 16, 61 ± 2 y)	Cardiac function (↑VO_2_max)
Sun et al., 2019 [46]	Twelve wk of low volume sprint interval training improves cardio-metabolic health outcomes in overweight females	RCT	12 wk, three sessions/week; HIIT: ~9 × 4 min cycling at 90% VO_2_peak + 3 min rest	Females with overweight status (*n* = 42, F): HIIT (*n* = 14, 21.5 ± 1.8 y), MICT (*n* = 14, 20.9 ± 1.4 y), and SIT groups (*n* = 14, 21.2 ± 1.4 y)	Cardiac function (↑VO_2_peak), blood biomarkers (↑insulin sensitivity and ↓fasting insulin), and body composition (↓BM)
de Lade et al., 2018 [47]	Effects of moderate intensity endurance training vs. high intensity interval training on weight gain, cardiorespiratory capacity, and metabolic profile in postnatal overfed rats	Animal Study	8 wk, three sessions/week; 40 min/session (10 min warm-up at 50% VO_2_max; 6 × 3 min at 85–90% VO_2_max, 2 min recovery at 50% VO_2_max)	Postnatal overfed rats (*n* = 80, M = 40, F = 40)	Cardiac function (↑VO_2_max), blood biomarkers (↑insulin sensitivity), and body composition (↓adiposity)
Lee et al., 2018 [37]	Effects of high-intensity interval training on vascular function in breast cancer survivors undergoing anthracycline chemotherapy: design of a pilot study	Pilot RCT	8 wk, three sessions/week; 7 × 1 min intervals at 90% PPO, interspersed with 2 min recovery at 10% PPO; 5 min warm-up/cool-down	Survivors of breast cancer (*n* = 30, F): HIIT (*n* = 15) and control groups (*n* = 15); >18 y	Cardiac function (↑VO_2_max), vascular function (↑FMD), and others (adherence)
Jurio-Iriarte and Maldonado-Martín, 2019 [30]	Effects of Different Exercise Training Programs on CRF in Overweight/Obese Adults With Hypertension: A Pilot Study	Single-blind RCT (pilot study)	8, 12, or 16 wk, two sessions/week; treadmill: 4 × 4 min at 76–95% HR reserve with 3 min recovery; bike: 30 s at high intensity followed by 60 s at moderate intensity, progressing to 18 repetitions	Individuals with hypertension and obesity: study 1 (8 wk, *n* = 18): control (*n* = 8), MICT (*n* = 6), and HIIT groups (*n* = 6); dropouts (*n* = 2); study 2 (12 wk, *n* = 26): control (*n* = 8), MICT (*n* = 10), and HIIT groups (*n* = 8); study 3 (16 wk, *n* = 20): control (*n* = 89), MICT (*n* = 7), and HIIT groups (*n* = 8); dropouts (*n* = 4); 55.9 ± 8.5 y	Cardiac function (↑VO_2_peak, ↑MET, ↑CPET, and MSWT distance)
Ingul et al., 2018 [40]	Effect of High Intensity Interval Training on Cardiac Function in Children with Obesity: A Randomised Controlled Trial	RCT	12 wk, three sessions/week; 4 × 4 min intervals at 85–95% HRmax, with 3 min active recovery; isocaloric to MICT	Children with obesity (*n* = 99, M/F): HIIT (*n* = 33, 7–16 y); MICT (*n* = 32, 7–16 y); nutrition groups (*n* = 34, 7–16 y)	Cardiac function (↑VO_2_peak and ↑LV function)
Adams et al., 2017 [28]	Effects of high-intensity aerobic interval training on CVD risk in testicular cancer survivors: A phase 2 randomized controlled trial	RCT	12 wk, three sessions/week; 4 × 4 min treadmill intervals (progressing from 75 to 95% VO_2_peak), 3 min active recovery at 5–10% below ventilatory threshold; total session time: 35 min	Survivors of testicular cancer (*n* = 63, 43.7 ± 10.8 y): HIIT (*n* = 35, 44.0 ± 11.6 y) and usual care groups (*n* = 28, 43.3 ± 9.9 y)	Cardiac function (↑VO_2_peak, ↓resting HR, and ↑HR recovery), vascular function (↑carotid distensibility, ↓CIMT, ↑brachial artery diameter, and ↓vascular age), and blood biomarkers (↓LDL-C and ↓hs-CRP)
Álvarez et al., 2017 [48]	Effects and prevalence of nonresponders after 12 wk of high-intensity interval or resistance training in women with insulin resistance: a randomized trial	RCT	12 wk, three sessions/week; progressive cycling HIIT with 12 work intervals (70–100% HRR); inactive recovery periods; total session time: 38 min	Women with insulin resistance (*n* = 35): HIIT (*n* = 18, 38 ± 8 y) and resistance training groups (*n* = 17, 33 ± 7 y)	Cardiac function (↓resting HR), blood biomarkers (↓SBP, ↓DBP, ↓fasting glucose, ↓insulin, ↓HOMA-IR), and body composition (↓fat mass, ↓WC, ↓skinfold thicknesses)
Ellingsen et al., 2017 [29]	High-Intensity Interval Training in Patients With Heart Failure With Reduced Ejection Fraction	RCT	12 wk, three sessions/week; 4 × 4 min intervals at 90–95% HRmax, interspersed with 3 min active recovery; total session time: 38 min including warm-up and cool-down	Patients with congestive heart failure (*n* = 215, M/F): HIIT (*n* = 77, M = 63, F = 14, 55-68 y); MCT (*n* = 65, M = 53, F = 12, 58-65 y); resistance and rehabilitation exercise groups (*n* = 73, M = 59, F = 14, 55–65 y)	No significant change
Toohey et al., 2016 [49]	A pilot study examining the effects of low-volume high-intensity interval training and continuous low to moderate intensity training on QoL, functional capacity and CV risk factors in cancer survivors	Pilot RCT	12 wk, three sessions/week; 7 × 30 s intervals at ≥85% HRmax, 1 min rest between intervals; 5 min warm-up and cool-down; progressive increase in intervals over first five sessions	BC survivors (*n* = 16, F): LVHIIT (*n* = 8) and CLMIT groups (*n* = 8); 51.6 ± 13.01 y	Cardiac function (↑6MWT distance), blood biomarkers (↓SBP, ↓DBP, ↓MAP, ↓CSP, ↓PP, ↓AP, ↓CDP), body composition (↓Hip, ↓Waist), and others (↑QoL)
Weston et al., 2016 [44]	Effect of Novel, School-Based High-Intensity Interval Training(HIT) on Cardiometabolic Health in Adolescents: Project FFAB(Fun Fast Activity Blasts)—An Exploratory Controlled Before-And-After Trial	Controlled before-and-after study	10 wk, three sessions/week: 4–7 × 45 s maximal effort drills (basketball, boxing, dance, soccer) at ≥90% HRmax, interspersed with 90 s recovery	Adolescents (*n* = 101, M): control (*n* = 60) and HIIT groups (*n* = 41); 14.1 ± 0.3 y	Cardiac function (↑ 20m shuttle run test), blood biomarkers (↓TG), body composition (↓WC), and others (↑MVPA levels)
Hwang et al., 2016 [25]	Novel all-extremity high-intensity interval training improves aerobic fitness, cardiac function and insulin resistance in healthy older adults	RCT	8 wk, 4 days/week of all-extremity ergometer exercise. HIIT: 4 × 4 min at 90% HRpeak, interspersed by 3 × 3 min active recovery at 70% HRpeak (25 min total). A 10 min warm-up and a 5 min cool-down at 70% HRpeak were included	Older adults with T2DM (*n* = 43, M/F): HIIT (*n* = 15, 64.8 ± 1.4 y), MICT (*n* = 14, 65.6 ± 1.8 y), and control groups (*n* = 16, 63.8 ± 1.6 y)	Cardiac function (↑VO_2_peak and ↑EF), vascular function (↑endothelial function), and blood biomarkers (↓insulin resistance)
Jaureguizar et al., 2016 [27]	Effect of High-Intensity Interval Versus Continuous Exercise Training on Functional Capacity and QoL in Patients With Coronary Artery Disease: A RANDOMIZED CLINICAL TRIAL	RCT	8 wk, three sessions/week on a cycle ergometer (40 min total). First month: 20 s peak intervals at 50% of max workload (SRT), followed by 40 s recovery at 10%. S month: intensity adjusted based on a new SRT (peak intervals)	Patients with CAD (*n* = 72, M): HIIT (*n* = 36, 58 ± 11 y) and MCT groups (*n* = 36, 58 ± 11 y)	Cardiac function (↑VO_2_peak, ↑6MWT distance, and ↑HR recovery) and others (↑QoL)
Higgins et al., 2015 [45]	Heterogeneous responses of personalised high intensity interval training on type 2 diabetes mellitus and CVD risk in young healthy adults	Clinical yrial	6 wk, three sessions/week on a cycle ergometer: 5 min warm-up, 3 × 1 min maximal intensity intervals (>120 rpm) at breaking wattage, 2 min working recovery, 3 min cool-down. Wattage adjusted by 10% based on performance and effort	Patients with T2DM (*n* = 23): HIIT (*n* = 13) and control groups (*n* = 10); sex and age not specified	Cardiac function (↑VO_2_max) and vascular function (↓SBP and ↓DBP)

Abbreviations: 6MWT—6 min walk test; ABI—ankle–brachial index; AP—augmented pressure; AWT—anterior wall thickness; BFM—body fat mass; Bcl-2—B-cell lymphoma 2; BMI—body mass index; BM—body mass; CAD—coronary artery disease; CDP—central diastolic pressure; CK-MB—creatine kinase myocardial band; CIMT—carotid intima-media thickness; CLMIT—continuous low- to moderate-intensity training; CPET—cardiopulmonary exercise test (distance); c-Myc—cellular myelocytomatosis oncogene; DBP—diastolic blood pressure; DFT—diastolic filling time; EF—ejection fraction; ESV—end-systolic volume; FBG—fasting blood glucose; FEV1—forced expiratory volume in 1 s; FMD—flow-mediated dilation; GLUT-4—glucose transporter type 4; GSK3B—glycogen synthase kinase-3 beta; HbA1c—hemoglobin A1C; HDL-C—high-density lipoprotein cholesterol; HOMA-IR—homeostatic model assessment for insulin resistance; hs-CRP—high-sensitive C-reactive protein; IMTG—intramuscular triglyceride; LDH—lactate dehydrogenase; LDL-C—low-density lipoprotein cholesterol; LV—left ventricular; LVHIIT—low-volume high-intensity interval training; MAP—mean arterial pressure; MET—metabolic equivalent; MSWT—modified shuttle walk test (distance); MV—min ventilation; MVPA—moderate-to-vigorous physical activity; PWT—posterior wall thickness; PP—pulse pressure; PWV—pulse wave velocity; QoL—quality of life; RHR—resting heart rate; RV—right ventricular; SBP—systolic blood pressure; SOCS3—suppressor of cytokine signaling 3; SV—stroke volume; T2DM—type 2 diabetes mellitus; T3—triiodothyronine; TG—triglyceride; TSH—thyroid-stimulating hormone; VO_2_max—maximal oxygen consumption; WC—waist circumference; ↑—increase; ↓—decrease.

## Data Availability

The original contributions presented in this study are included in this article. Further inquiries can be directed to the corresponding author.
